# Explainable Deep–Shallow Feature Fusion of Two-Dimensional Encoded Vis–NIR Spectra and RGB Image Features for Chilled Lamb Freshness Assessment

**DOI:** 10.3390/foods15142538

**Published:** 2026-07-17

**Authors:** Yanjie Ren, Qi Zhang, Yongqian Zhou, Hanwen Chen, Doudou Zhang, Zhigang Li, Peilin Jin

**Affiliations:** 1 College of Information Science and Technology, Shihezi University, Shihezi 832003, China; renyanjie@stu.shzu.edu.cn (Y.R.); m17599933929@163.com (Y.Z.); 20242108049@stu.shzu.edu.cn (H.C.); zhangdoudou@stu.shzu.edu.cn (D.Z.); 2 Xinjiang Production and Construction Corps Key Laboratory of Computing Intelligence and Network Information Security, Shihezi University, Shihezi 832003, China; zhangqi026@stu.shzu.edu.cn; 3 College of Mechanical and Electrical Engineering, Shihezi University, Shihezi 832003, China

**Keywords:** chilled lamb, freshness classification, two-dimensional encoding, feature optimization, feature fusion

## Abstract

Quality deterioration of chilled lamb during storage poses a challenge to meat quality and safety control, making rapid and accurate freshness-grade classification essential. Existing methods based on either spectral information or RGB image information alone are insufficient to simultaneously characterize internal chemical changes and external appearance changes during lamb quality deterioration. To address this issue, this study developed a chilled lamb freshness-grade classification method by integrating deep features from two-dimensional visible–near-infrared (Vis–NIR) spectral encoding with RGB image features. In this method, one-dimensional Vis–NIR spectra were transformed into two-dimensional encoded images using Gramian angular difference field (GADF), Gramian angular summation field (GASF), Markov transition field (MTF), and recurrence plot (RP) to enhance the representation of inter-wavelength structural relationships in spectral sequences, thereby compensating for the limited ability of conventional one-dimensional spectral modeling to capture global correlations and local variation information. Meanwhile, recursive feature elimination (RFE)-selected spectral deep features were fused with Spearman-selected RGB image features to construct a deep–shallow classification model. The results showed that the fusion models outperformed the single-modality models, with GADF(10%)+Image-SVM achieving the best performance, yielding an accuracy, F1-score, and MCC of 0.966, 0.957, and 0.946, respectively. Shapley additive explanations (SHAP) analysis further indicated that GADF deep features were the primary contributors, while RGB image features provided effective complementary information, demonstrating the potential of the proposed method for rapid and nondestructive freshness-grade classification of chilled lamb.

## 1. Introduction

Lamb is valued for its high nutritional value and desirable flavor, but its rich protein and nutrient contents also make it susceptible to microbial growth and quality deterioration during storage. Microbial proliferation, endogenous enzymatic activity, and lipid oxidation can promote the degradation of proteins and lipids, generating metabolites such as ammonia, amines, aldehydes, and sulfides, which lead to changes in color, odor, texture, and eating quality [1,2]. Therefore, accurate assessment of lamb freshness is important for meat quality control and consumer safety assurance. During meat storage, protein degradation produces volatile basic nitrogenous compounds, commonly characterized as total volatile basic nitrogen (TVB-N). TVB-N has been widely used as a biomarker of protein and amine degradation and is commonly employed to evaluate meat freshness and spoilage degree [3]. However, conventional TVB-N determination usually requires chemical reagents, complex sample pretreatment, and laboratory operations, making it time-consuming, destructive, and difficult to apply for rapid monitoring during cold-chain storage, transportation, and sales [4,5].

In recent years, rapid and nondestructive detection techniques, including hyperspectral imaging [6], Raman spectroscopy [7], electronic nose technology [8], and near-infrared spectroscopy, have been increasingly applied to meat freshness evaluation. Although hyperspectral imaging and Raman spectroscopy have strong information representation capabilities, they are often limited by high equipment costs, complex data processing, and high operator training requirements [9]. Electronic nose systems can respond to volatile compounds generated during meat storage, but their signals are susceptible to environmental conditions, baseline drift, and cross-sensitivity [10,11]. In contrast, near-infrared spectroscopy is rapid, requires minimal sample pretreatment, and can reflect changes in chemical components such as moisture, protein, and fat, showing considerable potential for meat freshness assessment [12]. Previous studies have combined near-infrared or visible–near-infrared (Vis–NIR) spectroscopy with chemometric, machine learning, lightweight deep learning, and interpretable learning methods for TVB-N prediction and freshness-grade classification in meat products [13,14,15,16,17]. However, conventional one-dimensional spectral modeling is affected by peak overlap, scattering interference, and redundant variables, and its ability to exploit inter-wavelength structural relationships and deep discriminative information remains limited.

To overcome the limited structural representation of one-dimensional spectral modeling, transforming spectra into two-dimensional images has become a promising strategy. Deep learning models, especially convolutional neural networks (CNNs), can automatically extract local textures, spatial relationships, and high-level semantic features from image-like inputs. In time-series analysis, methods such as the Gramian angular summation field (GASF), the Gramian angular difference field (GADF), the Markov transition field (MTF) [18], and the recurrence plot (RP) [19] have been used to transform one-dimensional sequences into two-dimensional representations. Since Vis–NIR spectra are ordered wavelength-dependent signals, these methods can also be introduced into spectral analysis to enhance the representation of inter-wavelength relationships. Recent studies have applied Gramian angular field (GAF)-based or other two-dimensional representations to different types of ordered data. For spectral data, these methods have been used for soil nutrient prediction [20], moldy-core detection in apples [21], microplastic analysis [22], and wine variety traceability [23]. For sensor time-series data, Zhu et al. [24] converted gas-sensor time-series data into GAF images and employed a depth-wise separable convolutional neural network to achieve over 90% average classification accuracy on two public gas datasets. These studies demonstrate the feasibility of converting one-dimensional ordered signals into two-dimensional representations for deep feature extraction. However, end-to-end deep learning models may suffer from overfitting and limited generalization in small-sample tasks. Therefore, hybrid modeling strategies that combine deep feature extraction with machine learning classifiers have been increasingly adopted to improve model stability. Xu et al. [25] combined a dual-branch hyperspectral feature extraction network with partial least squares regression (PLSR) and support vector regression (SVR) for pork freshness prediction; Ji et al. [26] integrated CNN-CBAM-extracted features with support vector machine (SVM) for syrup adulteration identification in wolfberry honey; and Zhao et al. [27] combined DSC-SqueezeNet-extracted acoustic image features with extreme gradient boosting (XGBoost) for moldy pear core detection. These studies indicate that hybrid strategies can exploit deep feature representations while improving classification stability under small-sample conditions.

However, changes in lamb freshness are reflected not only in internal chemical components, such as moisture, protein, and fat, but also in external appearance attributes, including surface color, texture, and gloss. RGB image-based computer vision can extract color, texture, and morphological information from meat surfaces and has been used to evaluate meat freshness and quality. Taheri-Garavand et al. [28] combined computer vision features with a genetic algorithm and artificial neural network for real-time chicken freshness evaluation; Asmara et al. [29] used color histograms and texture descriptors, including gray-level co-occurrence matrix (GLCM), Gabor, and histogram of oriented gradients (HOG), for chicken freshness identification; and Sutarman et al. [30] used RGB color moment features and support vector machine (SVM) for chicken freshness discrimination. These studies indicate that RGB color and texture features can reflect apparent quality changes in meat, but they mainly characterize external visual information and are limited in capturing internal chemical changes. Therefore, single-modal information may be insufficient to comprehensively describe meat quality deterioration during storage. To overcome this limitation, multimodal information fusion has been applied to the detection of meat quality and freshness. Previous studies have fused spectral information with image texture features to predict beef freshness-related indicators and storage days, or combined spectral features with GLCM texture features for freshness discrimination of small yellow croaker [31,32]. Spectral–texture fusion has also been used to detect quality indicators such as myoglobin content in marinated lamb and characteristic amino acid content in beef [33,34].

Although two-dimensional spectral transformation and spectral-image information fusion have been increasingly investigated, several limitations remain for chilled lamb freshness assessment. Existing studies have mainly focused on one-dimensional spectral modeling, single spectral-modality two-dimensional encoding, or the direct fusion of conventional spectral features with image color and texture features. In contrast, limited attention has been paid to hybrid modeling strategies that combine deep features extracted from two-dimensional encoded Vis–NIR spectra with selected RGB color and texture features for chilled lamb freshness-grade classification. Moreover, the relative contributions of spectral deep features and RGB image features remain insufficiently interpreted. To address these limitations, chilled lamb was used as the research object in this study, and a deep–shallow feature fusion framework was developed by integrating two-dimensional encoded Vis–NIR spectral deep features with RGB image features for freshness-grade classification. Specifically, this study includes the following four aspects:

1. GADF, GASF, RP, and MTF were introduced to transform one-dimensional Vis–NIR spectra into two-dimensional encoded images, thereby enhancing the representation of inter-wavelength structural relationships.
2. A pretrained ResNet18 network was used as a deep feature extractor to obtain discriminative spectral deep features from the two-dimensional encoded Vis–NIR images, and the classification performance of different encoding methods was systematically compared.
3. The deep spectral features extracted from the optimal two-dimensional encoding method were further selected and fused with selected RGB color and texture features to construct a compact deep–shallow feature representation. The fused features were subsequently input into machine learning classifiers for chilled lamb freshness-grade classification.
4. SHAP analysis was used to quantify the relative contributions of spectral deep features and RGB image features in the optimal fusion model, thereby improving the interpretability of the multimodal classification results.

## 2. Materials and Methods

**Figure 1:**
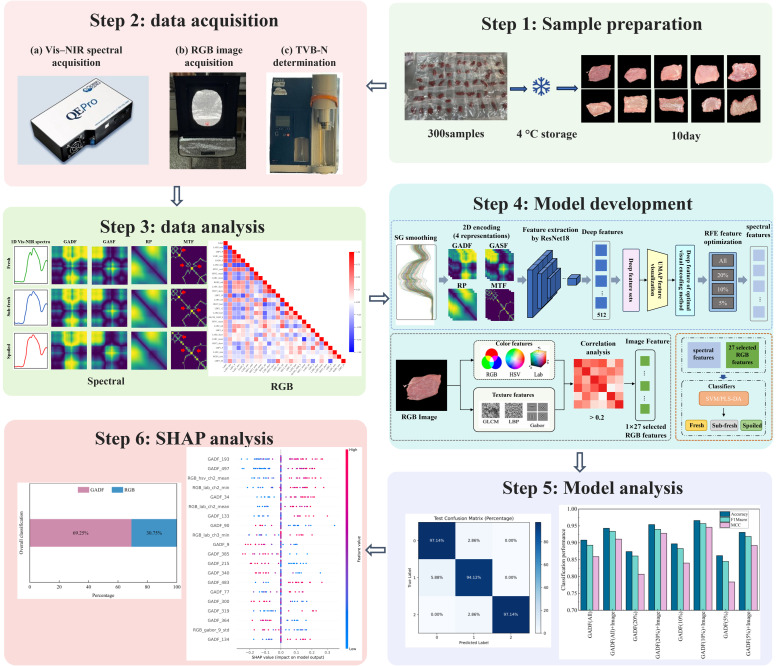
Flowchart of the main steps of data analysis and model establishment.

This study mainly consisted of six steps: sample preprocessing, data acquisition, data analysis, model development, result analysis, and SHAP analysis. The overall workflow is shown in Figure 1.

### 2.1. Sample Preparation

In this study, fresh hind leg meat purchased from five sheep at Jiuding Market in Shihezi, Xinjiang, China, was used as the experimental material. After purchase, the samples were immediately placed in an insulated container at 0–4 °C and transported to the laboratory. Visible surface fat and connective tissue were then removed using a sterile knife. The lamb samples from the five sheep were uniformly cut into 300 pieces in total, with 60 pieces obtained from each sheep. Each piece measured 80 mm × 60 mm × 8 mm and weighed approximately 20 ± 1 g. Each sample was individually sealed in a polyethylene bag and stored at 4 °C for 10 days.

For the storage experiment, 10 sampling points were established at 1-day intervals. Each day, six samples were randomly selected from the samples of each sheep, resulting in a total of 30 samples per day for subsequent experimental analysis. After continuous sampling for 10 days, a total of 300 samples were obtained and used for RGB image acquisition, spectral acquisition, and subsequent freshness analysis.

### 2.2. Data Acquisition and Preprocessing

#### 2.2.1. Spectral Data Acquisition and Preprocessing

**Figure 2:**
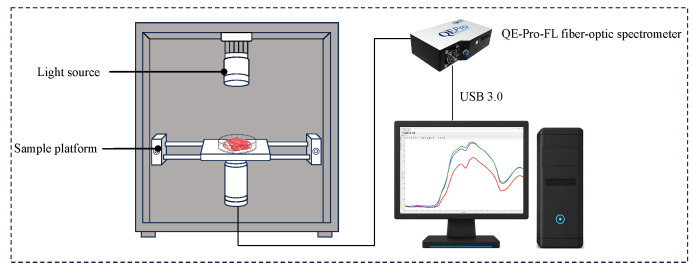
Visible–near-infrared (Vis–NIR) spectroscopy system.

Spectral data of the lamb samples were acquired using a Vis–NIR spectroscopy system, as shown in Figure 2. The spectral detection system consisted of a spectrometer, an optical fiber, a 74UV collimating lens, and a halogen lamp. In this study, we used a QE Pro-FL miniature fiber optic spectrograph (Ocean Insight, Inc., Dunedin, FL, USA) for spectral acquisition, with a wavelength range of 350–1100 nm and a spectral resolution of 0.69 nm. A halogen lamp (MR16, Signify (China) Investment Co., Ltd., Shanghai, China) was used as the light source, and the 74UV collimating lens was used to collect the light scattered by the sample. The collimating lens was connected to a fiber optic probe (QP600-2-VIS-NIR-OOS-00-5172-11, Ocean Insight, Inc., Dunedin, FL, USA) through a standard SMA905 interface. The collected spectral signals were transmitted to the spectrometer through the optical fiber for processing. Spectral data were acquired using OceanView 1.6.7 software (Ocean Insight, Inc., Dunedin, FL, USA) and transferred in real time to a computer through a USB 3.0 interface for storage. Before data acquisition, the detection system was warmed up for 30 min, followed by dark-current correction and reference correction. Before spectral measurement, the lamb samples were removed from the refrigerator at 4 °C and equilibrated under laboratory conditions of approximately 25 °C and 30 ± 5% relative humidity for 30 min to reduce the influence of temperature differences on spectral acquisition. During spectral acquisition, each lamb sample was placed flat on a fixed sample holder. The halogen lamp was positioned approximately 20 cm above the sample surface and illuminated the sample vertically. The distance between the fiber-optic probe and the sample surface was maintained at approximately 7 cm. The spectral measurement was performed on the central region of each sample to reduce the influence of edge effects, visible fat, connective tissue, and local surface heterogeneity. During spectral acquisition, the integration time was set to 12 ms. Each sample was independently measured twice under the same acquisition conditions, and the two spectra were then averaged and used as the raw spectral data of each individual sample. Each acquired spectrum contained 1044 wavelength points, and 919 wavelength variables were retained after removing the edge bands with relatively low signal-to-noise ratios. The retained spectral data were organized in a sample-by-wavelength format for subsequent analysis. During measurement, the relative positions of the halogen lamp, fiber-optic probe, collimating lens, and sample were kept constant. In addition, the spectral acquisition system was equipped with a blacklight-shielding cover to minimize interference from ambient light. All spectral measurements were conducted under the same laboratory conditions and acquisition geometry to improve the consistency and reproducibility of spectral acquisition.

To reduce the interference of abnormal spectra with subsequent modeling results, outlier sample screening was first performed after raw spectral acquisition. The spectral distributions of the samples were examined using principal component analysis (PCA) score space and Mahalanobis distance. The raw spectral matrix was mean-centered before PCA, and the first four principal components, explaining 99.02% of the total variance, were retained for Mahalanobis distance calculation. Samples with a squared Mahalanobis distance $D^{2}>\chi_{0.975,4}^{2}=11.143$ were regarded as outliers. According to this numerical criterion, 12 outlier samples were removed from the original 300 samples to improve the consistency and reliability of the dataset. On this basis, Savitzky–Golay (SG) smoothing was applied for spectral preprocessing to reduce high-frequency random noise while preserving the overall shape, peak characteristics, and main variation trends of the spectral curves. Since the Vis–NIR spectra of lamb samples contain continuous responses across adjacent wavelengths, overly complex preprocessing may change the original spectral intensity distribution and local curve characteristics. Therefore, SG smoothing was selected as a relatively moderate preprocessing method to improve spectral signal quality while retaining the main spectral information for subsequent analysis and modeling.

#### 2.2.2. Image Data Acquisition and Preprocessing

RGB images of the lamb samples were captured in a dark box environment, as shown in Step 2 of Figure 1. The dark box was entirely covered with blacklight-shielding material, forming an enclosed imaging space that effectively blocks external ambient light and reduces the influence of stray light. During image acquisition, the lamb samples were placed on black cardboard at the bottom of the dark box. RGB images were captured using an iPhone 14 Pro Max (Apple Inc., Cupertino, CA, USA), which was fixed at the top of the dark box to ensure that the imaging distance and angle between the camera and the samples remained consistent throughout the experiment. RGB images were acquired using the rear camera at 2× magnification, corresponding to an equivalent focal length of 48 mm. The samples were illuminated by the fixed light source inside the dark box. The smartphone camera was positioned approximately 40 cm above the sample surface, and images were captured from a top-view perspective. One RGB image was collected for each lamb sample under the fixed imaging conditions. The RGB image of each sample was matched with its corresponding Vis–NIR spectrum according to the sample number. The acquired RGB images were used for subsequent image feature extraction and classification modeling analysis.

### 2.3. Determination of TVB-N Content

TVB-N content of lamb samples was determined according to the Chinese National Food Safety Standard GB 5009.228-2016 [35]. For each measurement, 10 g of a fully minced lamb sample was weighed and placed into a distillation tube, mixed with 50 mL of distilled water; distilled water without sample addition was used as the blank control. Subsequently, 1 g of magnesium oxide (Shandong Keyuan Biochemical Co., Ltd., Heze, China) was added to the distillation tube, and distillation was performed using a FOSS Kjeltec 8200 Kjeldahl nitrogen analyzer (FOSS Analytical A/S, Hillerød, Denmark). The main parameters of the instrument are listed in Table 1. Before distillation, 30 mL of boric acid solution (20 g·L^−1^), prepared using boric acid (Shandong Keyuan Biochemical Co., Ltd., Heze, China), was added to the receiving flask, and the distillation time was set to 180 s. After distillation, 10 drops of a mixed indicator were added to the receiving solution. The mixed indicator was prepared by combining one part of 1 g·L^−1^ methyl red ethanol solution, prepared using methyl red (Shandong Keyuan Biochemical Co., Ltd., Heze, China), with five parts of 1 g·L^−1^ bromocresol green ethanol solution, prepared using bromocresol green (Shanghai Macklin Biochemical Co., Ltd., Shanghai, China). The receiving solution was then titrated with 0.1 mol·L^−1^ standard sulfuric acid solution (Guangdong Xinchengyuan Technology Co., Ltd., Huizhou, China) until it turned purple, and the titration volume was recorded. The TVB-N content of the lamb samples was calculated using the following equation:

(1) $$X=\frac{(V_{1}-V_{2})\times c\times 14}{m}\times 100$$

where *X* represents the TVB-N content of the lamb sample, expressed as mg/100 g; $V_{1}$ and $V_{2}$ represent the volumes of standard sulfuric acid titration solution consumed by the experimental group and the blank control, respectively; *c* represents the concentration of the standard sulfuric acid titration solution, expressed in mol·L^−1^; and *m* represents the mass of the minced lamb sample, expressed in g.

### 2.4. Feature Extraction

#### 2.4.1. Spectral Feature Extraction

Spectra are one-dimensional sequential signals that can reflect the internal chemical composition of samples and their variations. When modeling is performed directly from one-dimensional spectra, the potential global correlations and local structural features embedded in spectral sequences are often difficult to fully exploit. To enhance the representational capacity of spectral information and fully leverage the advantages of convolutional neural networks for image feature extraction, one-dimensional spectra were transformed into two-dimensional image representations for subsequent deep feature extraction and classification modeling. Before two-dimensional encoding, the SG-smoothed spectral data were first normalized to reduce the influence of spectral intensity scale differences among samples on the encoding results. Subsequently, GASF, GADF, MTF, and RP methods were used to transform the one-dimensional spectral sequences into two-dimensional encoded images for subsequent deep feature extraction.

ResNet18 [36] is a classical convolutional neural network characterized by the introduction of residual connection structures, which can effectively alleviate gradient vanishing and network degradation during the training of deep networks, while maintaining feature extraction capability with relatively low model complexity and high computational efficiency. To fully exploit the deep discriminative information contained in the encoded spectral images, this study employed a pretrained ResNet18 network as a unified deep feature extractor to extract deep features from two-dimensional spectral-encoded images. Considering the relatively limited sample size in this study, training a deep network from scratch may lead to unstable training and overfitting. Therefore, in this study, a ResNet18 model pretrained on the ImageNet dataset [37] was used as a fixed feature extractor to extract deep representation information from two-dimensional spectral-encoded images. ImageNet is a large-scale visual recognition dataset widely used for pretraining convolutional neural networks. In the specific implementation, all two-dimensional spectral-encoded images were uniformly resized to 224 × 224 pixels before being input into ResNet18. Since the pretrained ResNet18 network requires input images with a three-channel structure, the two-dimensional encoded results were uniformly converted into three-channel images before being fed into the network. The ResNet18 model was initialized using pretrained weight files and used as a fixed feature extractor, with its network parameters kept unchanged during feature extraction. Subsequently, the final fully connected classification layer was removed, and the output of the global average pooling layer was extracted as a 512-dimensional deep feature vector.

#### 2.4.2. RGB Color and Texture Feature Extraction

Color and texture features are important components of RGB images and play a crucial role in describing the overall color changes, intensity relationships between adjacent pixels, and spatial distribution patterns on the sample surface. The surface of lamb contains abundant color and texture information, which may contribute positively to freshness identification. Therefore, this study further investigated the roles of color and texture features in RGB images of lamb samples. Specifically, the acquired RGB images were first preprocessed to obtain the lamb sample region. Each image was converted to a grayscale image, and threshold-based segmentation was applied to separate the lamb sample from the black background. Morphological opening and closing operations were then performed to refine the foreground mask. The largest connected component was retained as the sample region, and its bounding rectangle was used to crop the region of interest (ROI). The ROI mask was further applied to the original RGB image so that subsequent feature extraction was performed within the segmented lamb sample region. On this basis, color features were extracted from the RGB, HSV, and CIELAB color spaces, while texture features were extracted from grayscale ROI images using GLCM, LBP, and Gabor algorithms. For each color channel, statistical descriptors were calculated. For GLCM features, the grayscale ROI images were quantized to 32 gray levels, and the pixel offsets were set to 1, 2, and 3 at four angles of 0°, 45°, 90°, and 135°. For LBP features, uniform LBP was used, and the radius–sampling point pairs were set to (1, 8), (2, 16), and (3, 24), corresponding to 10, 18, and 26 histogram bins, respectively. For Gabor features, the filter frequencies were set to 0.1, 0.2, and 0.3, and the orientations were set to 0°, 45°, 90°, and 135°. The color and texture information on the lamb surface was comprehensively characterized from multiple dimensions, including color distribution, grayscale statistical properties, spatial dependence, local patterns, and frequency-domain structures. Furthermore, correlation analysis was performed to select effective image features.

### 2.5. Feature Fusion

The model construction workflow of this study is shown in Step 4 of Figure 1. First, the SG-smoothed spectra were transformed into two-dimensional encoded images using GASF, GADF, MTF, and RP, respectively, and then fed into a pretrained ResNet18 network to extract 512-dimensional deep features. Subsequently, uniform manifold approximation and projection (UMAP) visualization was used to compare the class distributions of different encoding methods, and RFE was applied to select the spectral deep features. Meanwhile, color and texture features were extracted from RGB images, and effective image features were selected using Spearman correlation analysis. Finally, the selected spectral features were fused with RGB image features and input into SVM and PLS-DA models to classify the freshness grades of chilled lamb. To avoid information leakage, RFE and Spearman correlation analysis were performed only on the calibration set, and the selected feature indices were then applied to the prediction set for final evaluation.

Since the deep features extracted from two-dimensional spectral-encoded images by the deep network have relatively high dimensionality, whereas the number of features extracted from RGB images is relatively small, directly concatenating these two types of features may substantially increase the dimensionality of the fused feature space, leading to the curse of dimensionality, accumulation of redundant information, and model overfitting. Therefore, before fusing the deep features with RGB image features, it is necessary to further select the high-dimensional spectral features to retain the key information most relevant to the classification task and reduce feature redundancy.

RFE [38] is a commonly used wrapper-based feature selection method. Its core idea is to iteratively evaluate feature importance using a classifier and progressively remove features with lower contributions, thereby obtaining an optimal feature subset. Since RFE was applied to the spectral deep features before feature fusion in this study, the original spectral deep feature matrix can be denoted as $X_{\mathrm{spectral}}=\left[x_{1},x_{2},\ldots ,x_{m}\right]\in R^{n\times m}$, where *n* denotes the number of samples and *m* denotes the dimensionality of the spectral deep features. The class label vector is denoted as $y={\left[y_{1},y_{2},\ldots ,y_{n}\right]}^{T}$. In the *t*-th iteration, the base learner is trained on the current spectral feature subset $F_{\mathrm{spectral}}^{\left(t\right)}$, and the importance weight vector of each feature is calculated as follows:

(2) $$W^{\left(t\right)}=\left[w_{1}^{\left(t\right)},w_{2}^{\left(t\right)},\ldots ,w_{m_{t}}^{\left(t\right)}\right]$$

where $m_{t}$ denotes the number of features retained at the *t*-th iteration, and $w_{j}^{\left(t\right)}$ denotes the importance score of the *j*-th feature. Subsequently, the spectral features are ranked according to their weights, and several features with the smallest weights are removed to obtain the feature subset for the next iteration:

(3) $$F_{\mathrm{spectral}}^{\left(t+1\right)}=F_{\mathrm{spectral}}^{\left(t\right)}\setminus \left\{x_{j}|w_{j}^{\left(t\right)}\in \min\left(W^{\left(t\right)}\right)\right\}$$

After multiple recursive iterations, the final retained spectral feature subset can be expressed as follows:

(4) $$F_{\mathrm{spectral}}^{*}=\left\{x_{1},x_{2},\ldots ,x_{k}\right\},\;k<m$$

where *k* denotes the dimensionality of the selected spectral features. Through the recursive process of training, ranking, elimination, and retraining, RFE can reduce the interference of irrelevant or redundant features on model performance while preserving the main discriminative information.

In the specific implementation, the 512-dimensional GADF deep features extracted by ResNet18 were standardized using StandardScaler before RFE. RFE was performed using a linear SVM estimator with C = 1.0, and the elimination step was set to 5 features per iteration. Different feature retention ratios, including 20%, 10%, and 5%, were further evaluated for subsequent classification and fusion modeling.

### 2.6. Classifier Construction

In the construction of classification models, two classifiers, namely support vector machine (SVM) and partial least squares discriminant analysis (PLS-DA), were selected for modeling and comparison.

SVM is a supervised classification method based on statistical learning theory. Its basic principle is to identify an optimal classification hyperplane in the feature space that maximizes the margin between classes, thereby effectively separating samples from different categories. For linearly inseparable problems, SVMs can map input features into a high-dimensional space via kernel functions, thereby improving the model’s ability to fit complex nonlinear classification boundaries. Because SVM exhibits good generalization performance under high-dimensional and small-sample conditions and is highly adaptable to data with high feature dimensionality, it has been widely used in classification tasks.

PLS-DA is a supervised discriminant method developed from partial least squares regression. It mainly achieves dimensionality reduction and classification of high-dimensional data by establishing the relationship between the independent variable matrix and the class-response matrix. In PLS-DA, class variables are usually encoded in binary or dummy-variable form, where “1” indicates that a sample belongs to a given class and “0” indicates that it does not belong to that class [39]. This method can extract latent variables while retaining the main information relevant to class discrimination, making it particularly suitable for analyzing high-dimensional data with strong correlations among variables.

### 2.7. Evaluation Metrics

After outlier removal, the remaining 288 samples were divided into three freshness grades according to their TVB-N contents. The dataset was then divided into calibration and prediction sets using a sample-wise stratified splitting strategy according to freshness grade. Specifically, samples within each freshness grade were allocated to the calibration and prediction sets at an approximate ratio of 7:3 to maintain a similar class distribution between the two sets. For each lamb sample, the corresponding Vis–NIR spectral data and RGB image features were assigned to the same subset to avoid data leakage between model calibration and prediction. The sample distribution is shown in Table 2. To improve the stability of model evaluation and reduce the influence of a single data split on performance assessment, five-fold cross-validation was performed on the calibration set for model training and parameter optimization. In this study, to evaluate the accuracy and stability of the qualitative classification models, overall accuracy, precision, recall, F1-score, and Matthews correlation coefficient (MCC) were calculated for both the calibration and prediction sets. The formulas are as follows:

(5) $$\begin{array}{ccc} Accuracy & =\frac{1}{N}\sum_{i=1}^{C}n_{ii},\;Precision & =\frac{1}{C}\sum_{i=1}^{C}P_{i} \end{array}$$

(6) $$\begin{array}{ccc} Recall & =\frac{1}{C}\sum_{i=1}^{C}R_{i},\;F1\text{-}score & =\frac{1}{C}\sum_{i=1}^{C}\frac{2P_{i}R_{i}}{P_{i}+R_{i}} \end{array}$$

(7) $$MCC=\frac{c\times s-\sum_{k=1}^{C}p_{k}t_{k}}{\sqrt{\left(s^{2}-\sum_{k=1}^{C}p_{k}^{2}\right)\left(s^{2}-\sum_{k=1}^{C}t_{k}^{2}\right)}}$$

(8) $$\begin{array}{ccc} P_{i} & =\frac{n_{ii}}{\sum_{j=1}^{C}n_{ji}},\;R_{i} & =\frac{n_{ii}}{\sum_{j=1}^{C}n_{ij}} \end{array}$$

where *C* denotes the number of classes, and C=3 in this study; *N* denotes the total number of samples; $n_{ii}$ denotes the number of samples in the *i*-th class that are correctly classified; $n_{ij}$ denotes the number of samples whose true class is the *i*-th class and whose predicted class is the *j*-th class; *c* denotes the sum of the diagonal elements of the confusion matrix; *s* denotes the total number of samples; and $p_{k}$ and $t_{k}$ denote the number of samples predicted as the *k*-th class and the number of samples truly belonging to the *k*-th class, respectively.

### 2.8. SHAP-Based Interpretability Analysis

The SHAP method [40] was used to analyze the contributions of input features in the optimal fusion model, thereby improving the interpretability of the classification results. SHAP is based on the concept of Shapley values in cooperative game theory and decomposes model predictions into the contribution values of individual input features, thereby quantifying the influence of different features on model outputs. By analyzing the SHAP values of different features, the way in which each feature affects the model output can be explained, thus providing an interpretation of the model. The sign of a SHAP value indicates whether the feature has a positive or negative effect on the prediction result, while its absolute magnitude reflects the degree of influence of that feature on the model output.

## 3. Results

### 3.1. Statistical Analysis of TVB-N

**Figure 3:**
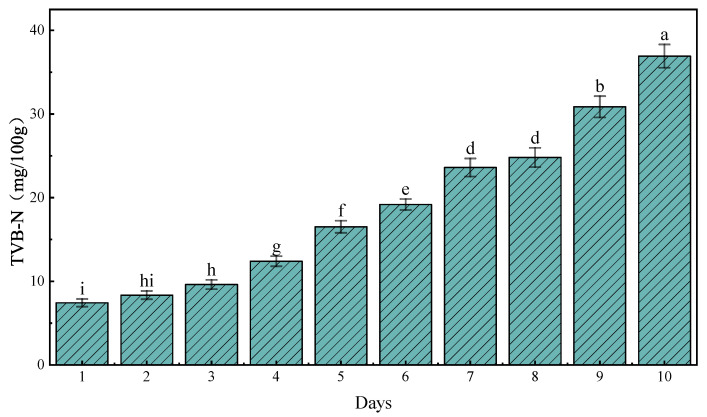
Changes in TVB-N content of lamb samples with storage time under refrigeration at 4 °C. Different lowercase letters indicate significant differences among groups at p<0.05.

TVB-N is a key indicator for evaluating meat freshness. As shown in Figure 3, the TVB-N content of chilled lamb showed a progressive and stage-dependent increase during refrigerated storage. According to previous studies [41,42] and Chinese National Food Safety Standards [35,43], meat with a TVB-N content below 15 mg/100 g is considered fresh, 15–20 mg/100 g is considered acceptable or sub-fresh, and values exceeding 20 mg/100 g indicate spoilage. From day 1 to day 4, the TVB-N content increased gradually but remained below 15 mg/100 g, indicating that the lamb samples were still in the fresh stage. Although a slight increase was observed during this period, the changes between some adjacent days were relatively small, suggesting that microbial activity and protein degradation were still at a relatively low level. On day 5, the TVB-N content increased significantly to 16.25 mg/100 g (p<0.05), exceeding the freshness threshold of 15 mg/100 g and indicating that the samples entered the acceptable or sub-fresh stage. With further extension of storage time, the TVB-N content continued to increase and reached 19.19 mg/100 g on day 6, approaching the spoilage threshold. From day 7 onward, the TVB-N content exceeded 20 mg/100 g, indicating that the samples entered the spoiled stage. Subsequently, the TVB-N content increased further, reaching 36.92 mg/100 g on day 10, clearly indicating severe spoilage of the lamb samples. This accelerated increase may be attributed to the continuous degradation of proteins and the accumulation of volatile alkaline nitrogenous compounds, such as ammonia and amines, during storage.

### 3.2. Spectral Analysis

#### 3.2.1. One-Dimensional Spectral Analysis

**Figure 4:**
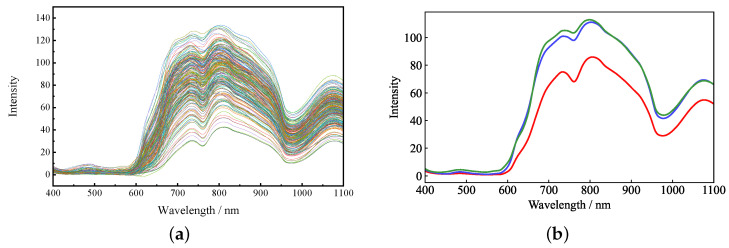
Vis–NIR spectral distributions of lamb samples with different freshness grades: (**a**) raw spectral curves of all samples; (**b**) mean spectral curves of samples with different freshness grades, where the red, blue, and green lines represent fresh, sub-fresh, and spoiled lamb samples, respectively.

All lamb samples were divided into three freshness grades according to their TVB-N contents. The average spectral curves of different freshness grades were then plotted, as shown in Figure 4. The overall variation trends of the spectral curves for different lamb samples were generally consistent, indicating that the samples had similar major chemical compositions and spectral response patterns. The spectral results showed that the main absorption bands of the lamb samples were observed at 450, 765, 830, and 970 nm. All samples exhibited an absorption valley near 450 nm, which may be attributed to vibrational absorption of O–H bonds in water molecules within the samples [44]. The absorption bands near 765 and 830 nm are typical absorption features of water molecules and are mainly associated with the second and third overtones of O–H stretching vibrations [45]. The absorption peak near 970 nm may be caused by stretching vibrations of functional groups such as C–H, C–O, and N–H in proteins, fats, and water [46].

#### 3.2.2. Comparison of Spectral Distributions Among Different Freshness Grades

**Figure 5:**
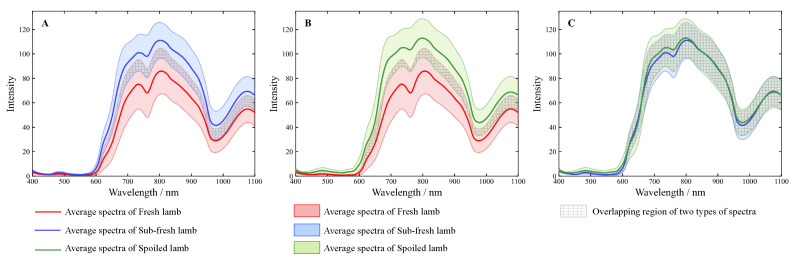
Pairwise comparison of spectral distributions of chilled lamb samples with different freshness grades: (**A**) Fresh vs. Sub-fresh samples; (**B**) Fresh vs. Spoiled samples; and (**C**) Sub-fresh vs. Spoiled samples. The gridded regions represent the overlapping portions of the spectral distributions between the two sample classes.

Figure 5 shows the pairwise spectral overlap among lamb samples with different freshness grades, where the gridded regions represent the overlapping parts of the spectra between two classes. As shown in the figure, spectral overlap existed among the three freshness grades of lamb samples. As shown in Figure 5A, fresh and sub-fresh samples exhibited obvious overlap across multiple wavelength ranges. In particular, their average spectral curves were relatively close in the main peak region and adjacent bands, indicating relatively small spectral differences between these two classes. As shown in Figure 5B, a certain degree of overlap was also observed between fresh and spoiled samples. However, compared with Figure 5A, the gridded overlap region was relatively smaller, suggesting that fresh and spoiled samples still showed more distinct spectral differences. In contrast, the overlap between sub-fresh and spoiled samples in Figure 5C was the most pronounced, with extensive gridded overlap in the main peak region, the descending region, and the subsequent rising region. This indicates that these two classes had higher similarity in their near-infrared responses and were the most difficult to distinguish.

These results indicate that although lamb samples with different freshness grades showed certain spectral differences, the overlap in their raw spectral distributions was relatively pronounced. The overlap was most evident between sub-fresh and spoiled samples, followed by that between fresh and sub-fresh samples, whereas fresh and spoiled samples were relatively easier to distinguish. This suggests that accurate identification of lamb freshness based solely on raw spectra remains a challenging task.

### 3.3. Analysis of Two-Dimensional Spectral-Encoded Images and Deep Feature Representations

#### 3.3.1. Visualization Analysis of Two-Dimensional Encoded Images

**Figure 6:**
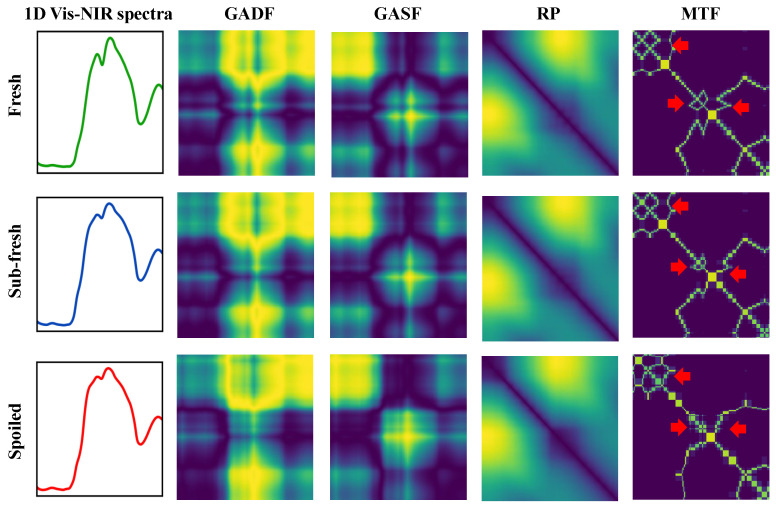
Visualization results of two-dimensional spectral encoding for lamb samples with different freshness grades (GADF: Gramian angular difference field; GASF: Gramian angular summation field; RP: recurrence plot; MTF: Markov transition field). In the 1D Vis–NIR spectra, the green, blue, and red lines represent fresh, sub-fresh, and spoiled lamb samples, respectively. The red arrows indicate the regions in the MTF images where the differences among freshness grades are more apparent.

Figure 6 presents the images obtained by applying two-dimensional encoding to the spectra of lamb samples with three different freshness grades. As shown in the figure, different encoding methods exhibited obvious differences in their ability to represent spectral information. Among them, the GADF images exhibited clearer structural differences among the three sample classes. In particular, distinct inter-class differences could be observed in the central region, the distribution of symmetric textures, and local variations in brightness, indicating that GADF can effectively map discriminative spectral information into a two-dimensional space. The GASF images also showed a strong symmetric structure, but the inter-class differences were mainly concentrated in the overall brightness distribution and variations in the central region. The overall texture of the RP images was relatively smooth, and only limited differences were observed among categories in certain regions, suggesting relatively weak inter-class separability. In the MTF images, differences among the three sample classes were mainly reflected in local regions. In particular, certain structural variations could be observed at the positions indicated by the red arrows in the figure; however, the overall discriminative information remained limited.

Overall, all four two-dimensional encoding methods enhanced the representational capacity of the original spectra to some extent, but each method emphasized different aspects of the feature information.

#### 3.3.2. Clustering Visualization Analysis of Deep Features from Different Encodings

**Figure 7:**
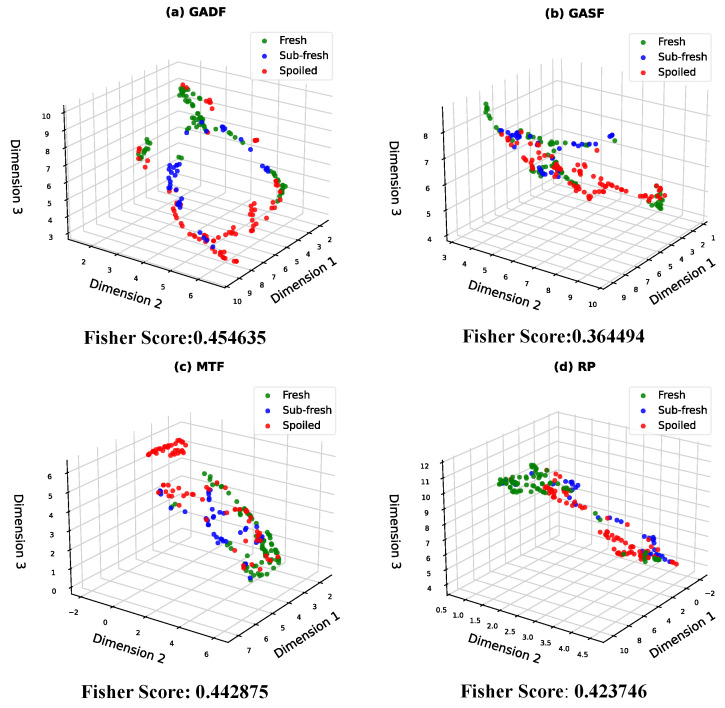
UMAP visualization results of deep features extracted by ResNet18 from different two-dimensional spectral encodings.

To further compare the effects of different two-dimensional encoding methods on the representational capability of spectral deep features, a pretrained ResNet18 model was used to extract deep features from GADF-, GASF-, MTF-, and RP-encoded images, and UMAP was employed to project the high-dimensional features into a 3D space for visualization analysis. Figure 7 shows the three-dimensional UMAP distribution results of the deep features corresponding to the four encoding methods, and the class discrimination capability was quantitatively evaluated using the Fisher score. The Fisher score measures feature discriminative ability by calculating the ratio of between-class variance to within-class variance, with a larger value indicating higher class separability [47].

As shown in Figure 7, the three classes of samples exhibited varying degrees of overlap under all four encoding methods, indicating that freshness changes in chilled lamb are continuous and that adjacent freshness grades are not completely discrete. As shown in Figure 7a, although local overlap was observed among the three classes in the GADF-encoded features, their overall distribution was relatively dispersed, with a clear inter-class separation and a low degree of overlap. As shown in Figure 7b, the three classes in the GASF encoded features were markedly mixed in the central region, and the class boundaries among fresh, sub-fresh, and spoiled samples were not sufficiently clear. As shown in Figure 7c, some spoiled samples formed a certain cluster in the MTF encoded features; however, obvious mixing among fresh, sub-fresh, and spoiled samples was still present in the central and lower-right regions. As shown in Figure 7d, the three classes in the RP encoded features generally exhibited a band-like distribution along a similar direction, with the most pronounced inter-class overlap and relatively weak discrimination.

Based on the UMAP visualization results and Fisher scores, GADF-encoded features performed relatively better in terms of class compactness, inter-class separation trends, and representation of continuous freshness changes. This may be because GADF places greater emphasis on the relative differences among wavelength points in the spectral sequence, while moisture migration, protein degradation, and lipid oxidation during lamb storage can alter the responses of specific bands and the relative relationships among wavelengths. Therefore, GADF is more conducive to capturing discriminative information related to freshness changes. Based on these results, GADF encoded images were selected for subsequent experiments in this study.

#### 3.3.3. Selection of Feature Extractor

To further compare the feature representation capabilities of different pretrained convolutional neural networks for GADF encoded images, ResNet18, SqueezeNet [48], and MobileNetV2 [49] were selected as candidate feature extractors. To ensure a fair comparison, all three networks were initialized with pretrained weights and used as fixed feature extractors, with their network parameters kept unchanged during feature extraction. All GADF-encoded images were uniformly resized to 224 × 224 pixels and converted to three-channel images through channel replication. Subsequently, deep features from the final feature layer of each network were extracted, and their classification performance was compared using the same classifier and evaluation metrics to determine the feature-extraction network for subsequent modeling. The classification results of different feature extractors are shown in Table 3.

As shown in Table 3, the classification performance of different pretrained feature extractors on GADF encoded images varied markedly. Among them, ResNet18 achieved the best performance, with all evaluation metrics being the highest among the three models. In particular, for the two key metrics of F1-score and MCC, ResNet18 outperformed SqueezeNet by 0.109 and 0.142, respectively, and MobileNetV2 by 0.086 and 0.125, respectively. This indicates that the deep features extracted by ResNet18 had greater advantages in balanced recognition across the three lamb freshness classes and in overall classification reliability. In contrast, SqueezeNet showed relatively lower overall performance, with an F1-score and MCC of 0.784 and 0.717, respectively, suggesting that its feature representation capability was relatively limited for the current GADF spectral-encoded image task. MobileNetV2 achieved an F1-score and MCC of 0.807 and 0.734, respectively. Its overall performance was slightly better than that of SqueezeNet but remained clearly lower than that of ResNet18. Notably, MobileNetV2 achieved a precision of 0.832, which was higher than that of SqueezeNet, indicating a certain degree of precision in some prediction results. However, its F1-score and MCC remained relatively low, suggesting insufficient overall recognition coverage and classification stability across different freshness classes.

Overall, the results in Table 3 indicate that ResNet18 performed best in terms of overall accuracy, balanced class recognition, and classification reliability. Therefore, ResNet18 was selected as the deep feature extractor for subsequent GADF encoded images, and the extracted deep features were used for subsequent feature selection and multimodal fusion modeling.

### 3.4. Analysis of RGB Features

#### 3.4.1. Changes in RGB Appearance Under Different Storage Times

**Figure 8:**
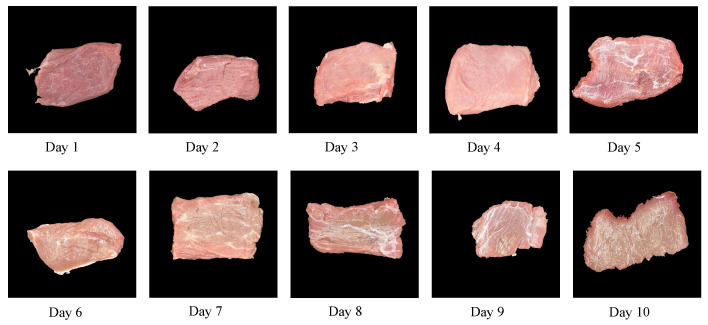
Changes in the RGB image appearance of lamb samples under refrigeration at 4 °C.

The appearance changes of lamb samples under different storage times are shown in Figure 8. As storage time increased, the lamb samples exhibited obvious time-dependent changes in color, surface condition, and tissue structure. During the early storage period Day 1–Day 4, the lamb samples showed relatively uniform coloration, ranging from bright red to light red. The surface gloss was relatively high, the meat structure remained intact, and the edge contours were clear. No obvious surface drying or exposure of the fibrous texture was observed, indicating that the overall appearance remained in a fresh state. As storage time extended to Day 5–Day 6, the color of the lamb samples gradually became lighter, changing from light red to pale pink. The surface gloss decreased, slight water loss appeared in local regions, subtle texture changes began to emerge on the meat surface, and the edge contours became slightly more irregular than those in the early storage period.

When the storage time was further extended to Day 7–Day 10, the appearance deterioration of the lamb samples became more pronounced. The overall color became markedly lighter and unevenly distributed, surface dryness intensified, fibrous textures were clearly exposed, local regions exhibited rough and uneven morphological characteristics, and the integrity of the tissue structure decreased considerably.

#### 3.4.2. RGB Feature Selection and Correlation Analysis

**Figure 9:**
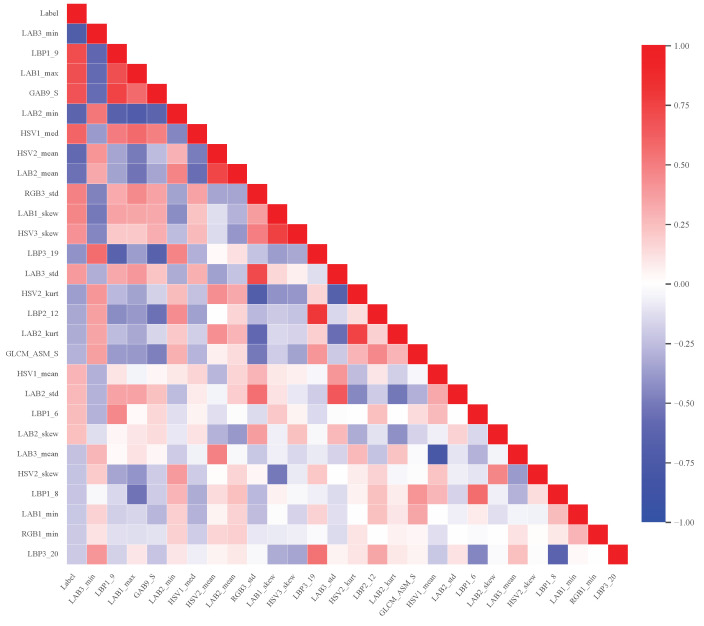
Spearman correlation coefficient matrix between RGB image features and class labels. The colors indicate the degree of correlation between the features and class labels: red represents positive correlation; blue represents negative correlation; and darker colors indicate stronger correlations.

Color and texture features were selected by calculating the Spearman rank correlation coefficients between RGB image features and class labels, with an absolute correlation coefficient greater than 0.2 used as the selection threshold. Finally, 27 image features were retained. Figure 9 shows the Spearman correlation coefficient matrix between the selected RGB features and freshness grades. The selected RGB features exhibited both positive and negative correlations with the class labels, indicating that different color and texture information showed differentiated responses during changes in lamb freshness.

Specifically, color-related features mainly characterize differences in surface brightness, chromaticity, and color distribution of lamb, which can reflect darkening, reduced gloss, and changes in surface condition during storage. Texture-related features mainly capture information such as local surface structures, grayscale spatial relationships, edge gradients, and texture roughness of lamb, which may be associated with tissue structural changes, surface dehydration, and texture deterioration during storage. These results indicate that the selected RGB features can comprehensively characterize changes in surface color and texture of lamb, providing effective image-based discriminative information for subsequent classification models.

### 3.5. Comparison of Classification Performance Between Single-Modal and Fusion Features

#### 3.5.1. Analysis of Classification Results Based on One-Dimensional Spectral Features and GADF Two-Dimensional Encoded Features

To verify the effectiveness of two-dimensional encoded deep features compared with conventional one-dimensional spectral modeling, the SG-smoothed one-dimensional spectra were used as baseline features, and SVM, PLS-DA, and K-nearest neighbor (KNN) were employed to construct classification models. Meanwhile, these results were compared with the classification results obtained using deep features extracted from GADF encoded images by ResNet18, as shown in Table 4.

As shown in Table 4, the models based on one-dimensional SG-NIR spectra already exhibited a certain capability for freshness classification, although their performance varied among different classifiers. Among the three one-dimensional spectral models, SG-NIR(1D)-PLS-DA achieved the best performance, with an accuracy, F1-score, and MCC of 0.885, 0.868, and 0.822, respectively. This indicates that PLS-DA has good adaptability to high-dimensional one-dimensional spectral variables and can extract effective information related to class discrimination through latent variables. SG-NIR(1D)-SVM achieved an accuracy, F1-score, and MCC of 0.874, 0.852, and 0.803, respectively, with overall performance slightly lower than that of PLS-DA. In contrast, SG-NIR(1D)-KNN showed relatively weak performance, with an accuracy, F1-score, and MCC of 0.805, 0.776, and 0.697, respectively. This may be because KNN is sensitive to distance metrics in high-dimensional spectral space and has difficulty forming stable class decision boundaries when sample distributions overlap.

Compared with direct modeling of one-dimensional spectra, the GADF two-dimensional encoded deep feature models showed better classification performance. Specifically, 2D-GADF-ResNet18-SVM achieved an accuracy, F1-score, and MCC of 0.908, 0.893, and 0.859, respectively. Compared with the best one-dimensional baseline model, SG-NIR(1D)-PLS-DA, these values increased by 0.023, 0.025, and 0.037, respectively. These results indicate that after transforming one-dimensional spectra into GADF two-dimensional encoded images, ResNet18 could further extract structured relationships and local variation information from the spectral sequences, thereby enhancing the model’s ability to discriminate lamb samples with different freshness grades.

#### 3.5.2. Analysis of Classification Results Based on RGB Image Features

The classification results of chilled lamb freshness based on all RGB image features and the selected RGB features are shown in Table 5. When all RGB image features were used, the PLS-DA model achieved an accuracy, F1-score, and MCC of 0.805, 0.726, and 0.691, respectively. After feature selection using Spearman correlation analysis, the corresponding metrics of the 27 selected RGB features increased to 0.816, 0.768, and 0.714, respectively. In comparison, the SVM model showed better overall performance. When all RGB image features were used, the SVM model achieved an accuracy, F1-score, and MCC of 0.816, 0.767, and 0.712, respectively. After feature selection, these three metrics further increased to 0.851, 0.814, and 0.767, respectively. These results indicate that Spearman-correlation-based feature selection can remove redundant and weakly correlated features while retaining color and texture features more closely associated with changes in lamb freshness, thereby improving the classification performance of RGB image features.

This performance improvement may be related to changes in appearance quality reflected in RGB image features. As storage time increases, the surface color, brightness, texture, and moisture status of lamb change. Color features can reflect differences in meat color, brightness, and color distribution, whereas texture features can describe surface structure, grayscale distribution, and local spatial variations. Therefore, the selected RGB image features can more specifically characterize appearance deterioration information during lamb storage. However, when relying solely on RGB image features, the classification performance of the model remained limited, because RGB images mainly reflect surface changes and are insufficient to fully characterize internal quality information such as protein degradation, moisture changes, and lipid oxidation. To further improve model classification performance, the 27 selected RGB image features were used as inputs for subsequent multimodal fusion.

#### 3.5.3. Analysis of Classification Results Based on Fusion Features

**Figure 10:**
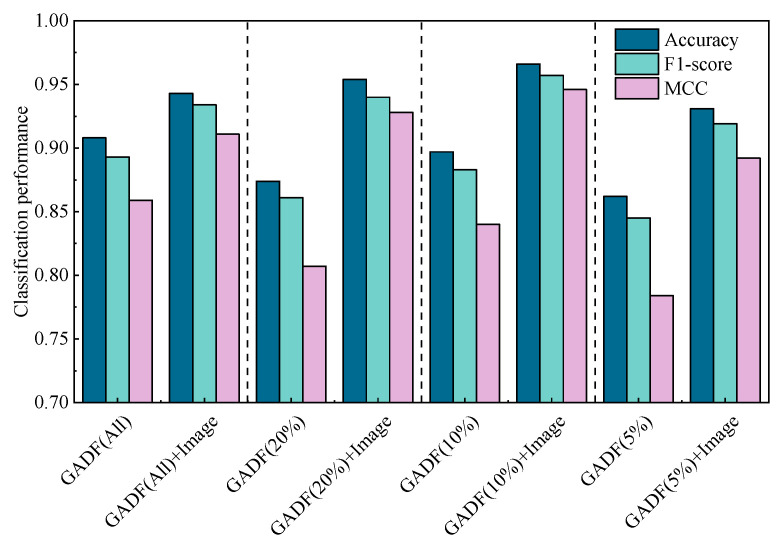
Comparison of SVM classification performance under different GADF feature retention ratios and their fusion with RGB image features.

**Figure 11:**
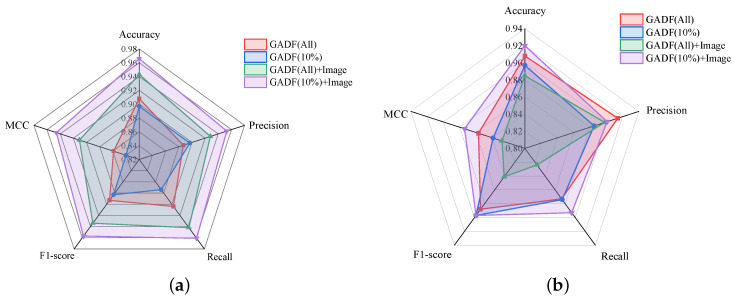
Radar charts of overall classification performance for representative GADF feature combinations and their fusion with RGB image features: (**a**) SVM; (**b**) PLS-DA.

To reduce redundant information in high-dimensional GADF deep features and enhance the effectiveness of feature representation in subsequent multimodal fusion, an RFE-based feature selection strategy was introduced after ResNet18 feature extraction. RFE recursively trains a base model and progressively eliminates features with lower contributions according to feature importance, thereby achieving discriminative feature selection and redundant dimensionality reduction. Based on this method, all GADF deep features were used as the initial feature set, and the top 20%, 10%, and 5% important GADF features were further selected and retained to compare the effects of different feature retention ratios on single-modal classification and RGB-fusion classification performance. Table 6 presents the classification results of SVM and PLS-DA under different feature combinations. Figure 10 visualizes the changes in three key metrics, namely accuracy, F1-score, and MCC, for the SVM models, while Figure 11 further compares the comprehensive performance of representative feature combinations across five evaluation metrics under the SVM and PLS-DA classifiers.

As shown in Table 6, the GADF single-modal features already demonstrated good capability for lamb freshness classification. When all GADF deep features were used for classification, the SVM model achieved an accuracy, F1-score, and MCC of 0.908, 0.893, and 0.859, respectively, indicating that the deep features extracted from GADF encoded images by ResNet18 could effectively characterize lamb freshness-related information. As the retention ratio of important GADF features gradually decreased, model performance showed certain fluctuations. When only the top 5% important GADF features were retained, the accuracy, F1-score, and MCC of the SVM model decreased to 0.862, 0.845, and 0.784, respectively, indicating that excessive compression of the feature space weakened the effective discriminative information in the spectral modality.

Compared with the use of GADF deep features alone, the overall model performance was markedly improved after fusing RGB image features. As shown in Table 6 and Figure 10, under the SVM classifier, the fusion of important GADF features retained at different ratios with RGB image features achieved higher accuracy, F1-score, and MCC than the corresponding GADF single-modal models. Among them, GADF(10%)+Image-SVM achieved the best classification performance, with an accuracy, F1-score, and MCC of 0.966, 0.957, and 0.946, respectively. Compared with the best spectroscopy-only model, GADF(All)-SVM, the optimal fusion model GADF(10%)+Image-SVM increased the accuracy, F1-score, and MCC from 0.908, 0.893, and 0.859 to 0.966, 0.957, and 0.946, respectively. In this optimal fusion model, 52 Vis–NIR spectral deep features selected by RFE and 27 RGB image features selected by Spearman correlation analysis were retained, forming a 79-dimensional multimodal feature vector for freshness classification. This feature composition reflects the independent feature selection results of the spectral and image modalities and provides a compact representation with reduced feature redundancy for subsequent classification. This indicates that appropriate feature selection can reduce redundant information, while the incorporation of RGB image features further enhances the separability of the fused feature space.

Figure 11 further shows the comprehensive performance of representative feature combinations under the SVM and PLS-DA classifiers. As shown in Figure 11a, under the SVM classifier, the GADF(10%)+Image model had the largest coverage area across the five evaluation metrics, indicating the best overall classification performance. As shown in Figure 11b, under the PLS-DA classifier, the fusion models also generally outperformed the corresponding single-modal models, but the improvement was smaller than that obtained with SVM, suggesting that PLS-DA had relatively limited adaptability to the current feature space.

Overall, GADF deep features effectively characterized spectral information related to lamb freshness; RFE-based feature selection reduced redundancy in high-dimensional features; and incorporating RGB image features further improved model classification performance. Among all models, GADF(10%)+Image-SVM achieved the best performance across all evaluation metrics, indicating that appropriate feature selection combined with RGB image feature fusion can effectively improve the discrimination ability for chilled lamb freshness grades.

#### 3.5.4. Bootstrap Confidence Interval Analysis of Representative Models

**Figure 12:**
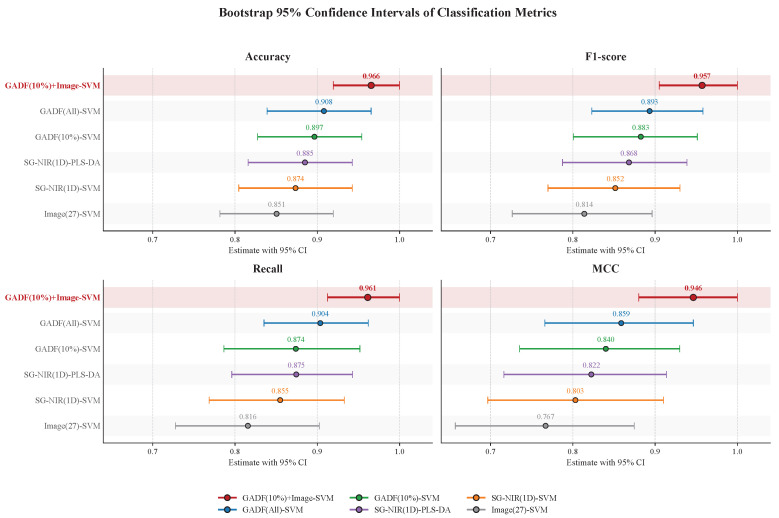
Bootstrap 95% confidence intervals of classification metrics for representative models. Point markers indicate the estimated metric values, and horizontal error bars represent the corresponding 95% bootstrap confidence intervals. Four evaluation metrics are shown, including Accuracy, F1-score, Recall, and Matthews correlation coefficient (MCC). The shaded row highlights the optimal GADF(10%)+Image-SVM model.

To further evaluate the uncertainty of the performance differences among different models, bootstrap confidence interval analysis was performed on the prediction-set results of representative models. The 95% bootstrap confidence intervals of Accuracy, F1-score, Recall, and MCC are shown in Figure 12. Overall, GADF(10%)+Image-SVM achieved the highest point estimates across all four metrics, with Accuracy, F1-score, Recall, and MCC values of 0.966, 0.957, 0.961, and 0.946, respectively. Compared with SG-NIR(1D)-SVM, SG-NIR(1D)-PLS-DA, Image(27)-SVM, GADF(All)-SVM, and GADF(10%)-SVM, the confidence intervals of GADF(10%)+Image-SVM were generally located in the higher-performance range.

Compared with the single-modal models, the optimal fusion model showed higher point estimates for all evaluation metrics, indicating that the fusion of selected GADF deep features and RGB image features improved the overall classification performance. Although partial overlap among the confidence intervals of some models was observed, GADF(10%)+Image-SVM consistently maintained the best performance across multiple evaluation metrics. These results indicate that the performance improvement of the proposed fusion model was not limited to a single metric, but was consistently reflected in overall accuracy, class-balanced recognition ability, and correlation-based classification reliability.

### 3.6. Classification Performance Analysis of the Optimal Fusion Model

**Figure 13:**
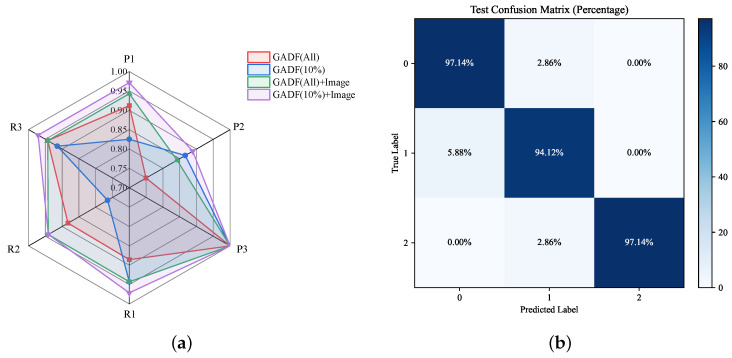
Class-wise precision and recall of representative models and confusion matrix of the optimal fusion model on the prediction set. (**a**) Radar chart of class-wise precision and recall for representative GADF feature combinations fused with RGB image features, where P1, P2, and P3 denote the precision of fresh, sub-fresh, and spoiled samples, respectively, and R1, R2, and R3 denote their corresponding recall values. (**b**) Percentage confusion matrix of the SVM model based on the fusion of the top 10% important GADF features and RGB image features on the prediction set. The horizontal axis represents the predicted classes, and the vertical axis represents the true classes.

To further analyze the recognition performance of the optimal fusion model for lamb samples with different freshness grades, an SVM model based on the fusion of the top 10% important GADF features and RGB image features was selected for in-depth analysis. Meanwhile, to compare the recognition balance of different representative models across categories, radar charts of class-wise precision and recall were plotted, and the percentage confusion matrix of the optimal fusion model on the prediction set was provided, as shown in Figure 13.

As shown in Figure 13a, when only all GADF deep features or the top 10% important GADF features were used, the models exhibited certain fluctuations in precision and recall for some classes, indicating that relying solely on deep features from two-dimensional spectral encoding was still insufficient for balanced recognition of samples with different freshness grades. After incorporating RGB image features, the GADF(All)+Image and GADF(10%)+Image models showed improved precision and recall for most classes, and the coverage area of the radar charts increased markedly, indicating that RGB image features could supplement information related to surface color and texture changes in lamb and thereby improve the class-balanced recognition ability of the model. Among them, the GADF(10%)+Image model showed the best overall performance across class-wise metrics, suggesting that the selected GADF deep features and RGB image features had better complementarity.

As shown in Figure 13b, the optimal fusion model achieved correct recognition rates of 97.14%, 94.12%, and 97.14% for fresh, sub-fresh, and spoiled samples, respectively, indicating high recognition performance for all three classes. A small number of sub-fresh samples were still misclassified, mainly as fresh samples, which may be because the sub-fresh stage represents an intermediate state during the transition from freshness to spoilage, during which its spectral responses and appearance features are similar to those of adjacent classes. Overall, the SVM model based on the fusion of the top 10% important GADF features and RGB image features not only achieved the best results in terms of overall accuracy, F1-score, and MCC, but also showed relatively balanced performance across different classes, enabling stable identification of lamb samples with different freshness grades. These results further validate the effectiveness of the proposed feature fusion scheme.

### 3.7. Interpretability Analysis of the Optimal Model

**Figure 14:**
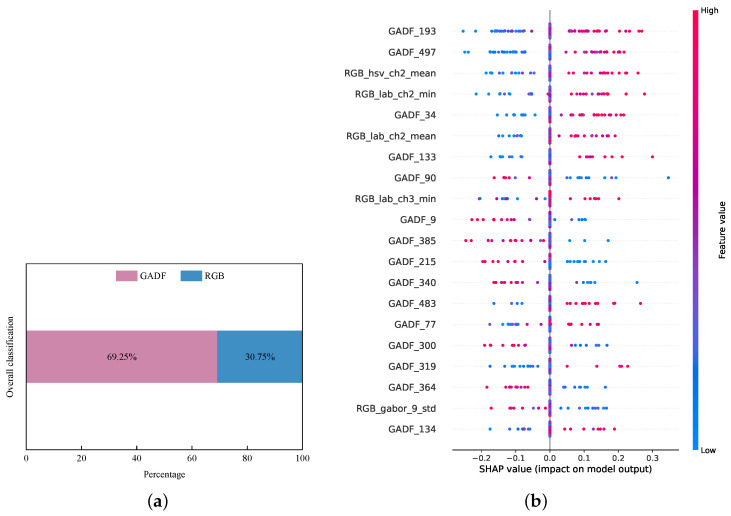
SHAP interpretability analysis results of the optimal fusion model. (**a**) Contribution proportions of GADF deep features and RGB image features to the overall classification results; (**b**) SHAP feature contribution distribution of the fused features.

To further explain the classification decision-making mechanism of the optimal fusion model, SHAP analysis was conducted on the prediction set based on the final trained SVM model with the selected fused features, as shown in Figure 14. As shown in Figure 14a, GADF deep features contributed 69.25% to the model output, whereas RGB image features contributed 30.75%. This indicates that, in the fusion model, GADF deep features remained the primary information source for lamb freshness classification. This is because spectra can reflect information related to internal quality changes in lamb during storage, and after GADF two-dimensional encoding and ResNet18-based deep feature extraction, discriminative spectral structural features can be generated. Meanwhile, the contribution of RGB features exceeded 30%, indicating that image information also played an important role in model discrimination and provided effective complementary information to the spectral deep features. As shown in Figure 14b, the top 20 high-contribution features included both GADF and RGB features, suggesting that the model did not rely on a single modality for classification, but instead integrated deep features from two-dimensional spectral encoding and RGB image features for discrimination.

The SHAP analysis indicated that the classification decisions of the SVM model based on the fusion of the top 10% important GADF features and RGB image features were mainly driven by GADF deep features while being complemented by RGB color and texture features. This further demonstrates that the fusion of GADF and RGB features can provide more comprehensive discriminative information for lamb freshness classification.

## 4. Discussion

### 4.1. Relationship Between Spectral–Image Features and Lamb Quality Changes

The results of this study showed that the fusion of two-dimensional Vis–NIR spectral encoding features and RGB image features improved the classification performance of chilled lamb freshness compared with single-modal models. This improvement can be attributed to the complementary information provided by the two modalities. Vis–NIR spectra mainly reflect internal chemical composition and its changes during storage, whereas RGB images mainly describe external appearance and surface structural information. Therefore, the fusion of spectral and image features provides a more comprehensive representation of lamb quality deterioration.

Among the different two-dimensional spectral encoding methods, GADF showed better feature representation capability, probably because it emphasizes the relative differences among wavelength points and captures changes in spectral response patterns associated with freshness deterioration. During storage, moisture migration, protein degradation, lipid oxidation, and the accumulation of volatile nitrogen compounds can affect the absorption responses of specific bands and the relationships among wavelengths; therefore, the selected GADF deep features were closely related to internal physicochemical changes in lamb. Meanwhile, the selected RGB image features mainly reflected external appearance and structural changes, including variations in surface color, brightness, gloss, texture distribution, local heterogeneity, and moisture-related surface conditions, which provided complementary information to the spectral features.

### 4.2. Comparison with Previous Studies

Compared with previous studies, the proposed GADF(10%)+Image-SVM model showed good performance in chilled lamb freshness classification. A Vis–NIR hyperspectral imaging method combined with RF achieved accuracies of 0.930 and 0.910 on the training and test sets for lamb freshness grade identification, respectively, while the full-wavelength PLS-DA model achieved a test-set accuracy of 0.850 [50]. Although these results demonstrated the feasibility of spectral methods, they still relied on conventional spectral representation and wavelength selection.

Deep learning-based spectral models have also been applied to meat freshness detection. A 1D-SE-ResNet model based on near-infrared spectroscopy achieved an accuracy of 0.937 for pork freshness detection [51], and CNN-based hybrid spectral models have also shown effectiveness in pork freshness detection [12]. However, these methods mainly model spectral information in a one-dimensional form or focus on a single spectral modality. In contrast, the present study introduced two-dimensional GADF encoding before deep feature extraction and further incorporated RGB image features, enabling the model to combine inter-wavelength structural information with surface color and texture information for chilled lamb freshness classification.

### 4.3. Limitations and Future Perspectives

Although the proposed model achieved satisfactory performance in chilled lamb freshness classification, this study still has some limitations. The current dataset was mainly obtained from a single market and may not fully cover the variability caused by differences in geographical origin, breed, supplier, and slaughter batch, which may limit model generalizability. In addition, the freshness labels were defined according to TVB-N thresholds, which cannot fully represent sensory attributes or microbiological safety. Future work will expand the sample sources, incorporate sensory evaluation, total viable count (TVC), and other microbiological indicators, and further validate the proposed method under real cold-chain monitoring and slaughterhouse inspection environments to improve its robustness and practical applicability.

## 5. Conclusions

In this study, a chilled lamb freshness classification method was developed by fusing deep features from two-dimensional Vis–NIR spectral encoding with RGB image features. One-dimensional spectra were transformed into two-dimensional encoded images using GASF, GADF, MTF, and RP, and deep features were extracted using a pretrained ResNet18 model. Among the four encoding methods, GADF demonstrated superior feature representation capability and was selected for subsequent feature optimization and fusion modeling. RFE-selected GADF deep features were further combined with Spearman-selected RGB image features to construct spectral-image fusion classification models.

The experimental results showed that the fusion models generally outperformed the single-modal models. Among them, the GADF(10%)+Image-SVM model achieved the best classification performance, with an accuracy, F1-score, and MCC of 0.966, 0.957, and 0.946, respectively. SHAP analysis further indicated that GADF deep features were the primary contributors to model discrimination, while RGB image features provided effective complementary information. These results demonstrate that fusing two-dimensional spectral encoding features with RGB image features is effective for rapid and nondestructive freshness classification of chilled lamb.

## Figures and Tables

**Table 1:** Main parameters of the FOSS Kjeltec 8200 instrument used for TVB-N determination.

Parameter	FOSS Kjeltec 8200
Measurement range	0.1–200 mg N
Sample weight range	0–50 g
Repeatability	0.01
Recovery rate	99.5%
Power	2200 W
Dimensions	480 mm × 580 mm × 690 mm

**Table 2:** Distribution of lamb samples in different freshness grades after outlier removal.

Freshness Grade	Storage Days	TVB-N Range	Total Samples	Calibration Set	Prediction Set
Fresh	Day 1–Day 4	<15 mg/100 g	115	80	35
Sub-fresh	Day 5–Day 6	15–20 mg/100 g	57	40	17
Spoiled	Day 7–Day 10	>20 mg/100 g	116	81	35
Total	–	–	288	201	87

**Table 3:** Comparison of classification performance among different pretrained feature extractors based on GADF two-dimensional encoded images. Note: bold values indicate the best performance for each metric.

Model	Accuracy	Precision	Recall	F1-Score	MCC
ResNet18	**0.908**	**0.887**	**0.904**	**0.893**	**0.859**
SqueezeNet	0.816	0.785	0.787	0.784	0.717
MobileNetV2	0.828	0.832	0.797	0.807	0.734

**Table 4:** Comparison of classification performance between one-dimensional spectra and GADF two-dimensional image encoded features. Note: bold values indicate the best performance for each metric, and the main hyperparameters of each classifier are also reported.

Feature Type	Feature Extractor	Classifier	Hyperparameters	Accuracy	Precision	Recall	F1-Score	MCC
2D-GADF	ResNet18	SVM	Kernel = RBF, C=4, γ=0.0004	**0.908**	0.887	**0.904**	**0.893**	**0.859**
2D-GADF	ResNet18	PLS-DA	LVs = 10	**0.908**	**0.914**	0.873	0.888	0.857
SG-NIR(1D)	–	SVM	Kernel = RBF, C=1, γ=0.006	0.874	0.849	0.855	0.852	0.803
SG-NIR(1D)	–	PLS-DA	LVs = 12	0.885	0.865	0.875	0.868	0.822
SG-NIR(1D)	–	KNN	k=6	0.805	0.779	0.778	0.776	0.697

**Table 5:** Comparison of classification performance for chilled lamb freshness using all RGB image features and selected RGB image features. Note: bold values indicate the best performance for each metric. The main hyperparameters of each classifier are also reported.

Features	Model	Hyperparameters	Accuracy	Precision	Recall	F1-Score	MCC
Image(All)	SVM	Kernel = RBF, C=0.8, γ=0.00515	0.816	0.767	0.767	0.767	0.712
Image(27)	SVM	Kernel = RBF, C=0.7, γ=0.027	**0.851**	**0.813**	**0.816**	**0.814**	**0.767**
Image(All)	PLS-DA	LVs = 3	0.805	0.736	0.727	0.726	0.691
Image(27)	PLS-DA	LVs = 7	0.816	0.774	0.767	0.768	0.714

**Table 6:** Comparison of classification performance under different GADF feature retention ratios and their fusion with RGB image features. Note: bold values indicate the best performance for each metric. The main hyperparameters of each classifier are also reported.

Features	Number	Model	Hyperparameters	Accuracy	Precision	Recall	F1-Score	MCC
GADF(All)	512	SVM	Kernel = RBF, C=4, γ=0.0004	0.908	0.887	0.904	0.893	0.859
		PLS-DA	LVs = 10	0.908	0.914	0.873	0.888	0.857
GADF(20%)	103	SVM	Kernel = RBF, C=4, γ=0.000368	0.874	0.853	0.875	0.861	0.807
		PLS-DA	LVs = 5	0.897	0.877	0.884	0.880	0.837
GADF(10%)	52	SVM	Kernel = RBF, C=8, γ=0.01	0.897	0.897	0.874	0.883	0.840
		PLS-DA	LVs = 5	0.897	0.885	0.874	0.897	0.839
GADF(5%)	26	SVM	Kernel = RBF, C=12, γ=0.002	0.862	0.845	0.845	0.845	0.784
		PLS-DA	LVs = 5	0.851	0.848	0.796	0.809	0.768
GADF(All)+Image	539	SVM	Kernel = RBF, C=4, γ=0.0014	0.943	0.928	0.942	0.934	0.911
		PLS-DA	LVs = 8	0.885	0.898	0.824	0.841	0.828
GADF(20%)+Image	130	SVM	Kernel = RBF, C=0.8, γ=0.009	0.954	0.951	0.932	0.940	0.928
		PLS-DA	LVs = 20	0.897	0.908	0.844	0.861	0.844
GADF(10%)+Image	79	SVM	Kernel = RBF, C=1, γ=0.0174	**0.966**	**0.953**	**0.961**	**0.957**	**0.946**
		PLS-DA	LVs = 20	0.920	0.901	0.893	0.897	0.874
GADF(5%)+Image	53	SVM	Kernel = RBF, C=3, γ=0.009	0.931	0.916	0.923	0.919	0.892
		PLS-DA	LVs = 20	0.908	0.908	0.863	0.875	0.857

## Data Availability

The data presented in this study are available on request from the corresponding authors. The data are not publicly available due to restrictions related to data ownership and ongoing research.

## References

[1] Liang C., Zhang D., Zheng X., Wen X., Yan T., Zhang Z., Hou C. (2021). Effects of different storage temperatures on the physicochemical properties and bacterial community structure of fresh lamb meat. Food Sci. Anim. Resour..

[2] Toomik E., Rood L., Bowman J.P., Kocharunchitt C. (2023). Microbial spoilage mechanisms of vacuum-packed lamb meat: A review. Int. J. Food Microbiol..

[3] Bekhit A.E.D.A., Holman B.W., Giteru S.G., Hopkins D.L. (2021). Total volatile basic nitrogen (TVB-N) and its role in meat spoilage: A review. Trends Food Sci. Technol..

[4] Zhang D., Zhu L., Jiang Q., Ge X., Fang Y., Peng J., Liu Y. (2023). Real-time and rapid prediction of TVB-N of livestock and poultry meat at three depths for freshness evaluation using a portable fluorescent film sensor. Food Chem..

[5] Gao H., Liu C., Mou L., Zhao M. (2025). Meat Freshness Evaluation Methods Based on Spectral Technology: From Traditional to Machine Learning-Enhanced Approaches: A Review. J. Food Saf. Food Qual.-Arch. Für Leb..

[6] Zhu R., Bai Z., Qiu Y., Zheng M., Gu J., Yao X. (2021). Comparison of mutton freshness grade discrimination based on hyperspectral imaging, near infrared spectroscopy and their fusion information. J. Food Process Eng..

[7] Liu Q., Dong P., Fengou L.C., Nychas G.J., Fowler S.M., Mao Y., Luo X., Zhang Y. (2023). Preliminary investigation into the prediction of indicators of beef spoilage using Raman and Fourier transform infrared spectroscopy. Meat Sci..

[8] Wei G., Lv X., Zhao J., Zhang W., Wang B., Dou Q., Zhang X. (2025). A prior knowledge boosted CNN-LSTM prediction model for oyster freshness evaluation based on an electronic nose. J. Food Compos. Anal..

[9] Qu C., Li Y., Du S., Geng Y., Su M., Liu H. (2022). Raman spectroscopy for rapid fingerprint analysis of meat quality and security: Principles, progress and prospects. Food Res. Int..

[10] Berna A. (2010). Metal oxide sensors for electronic noses and their application to food analysis. Sensors.

[11] Sanislav T., Mois G.D., Zeadally S., Folea S., Radoni T.C., Al-Suhaimi E.A. (2025). A comprehensive review on sensor-based electronic nose for food quality and safety. Sensors.

[12] Zhao X., Ning W., Chen R., Wang H., Zhang G., Bi J., Hou H. (2025). Rapid non-destructive detection of pork freshness using visible-near infrared spectroscopy based on convolutional neural network hybrid models. J. Food Compos. Anal..

[13] Zhang F., Kang T., Sun J., Wang J., Zhao W., Gao S., Wang W., Ma Q. (2022). Improving TVB-N prediction in pork using portable spectroscopy with just-in-time learning model updating method. Meat Sci..

[14] Li X., Wei C., Liang B. (2024). Near-Infrared Spectroscopy-Based Chilled Fresh Lamb Quality Detection Using Machine Learning Algorithms. J. Food Saf..

[15] Wu N., Zhang J., Wang Q., Guan X., Zhang L. (2025). Rapid detection of mutton freshness grades and TVB-N using hyperspectral imaging with optimized machine learning and lightweight deep learning models. J. Food Compos. Anal..

[16] Zhai C., Jiang X., Xu Z., Wudan M., Li D. (2025). Non-destructive detection of chilled mutton freshness using a dual-branch hierarchical spectral feature-aware network. Foods.

[17] Chen R., Ning W., Xie X., Bi J., Zhang G., Hou H. (2026). Non-Destructive Assessment of Beef Freshness Using Visible and Near-Infrared Spectroscopy with Interpretable Machine Learning. Foods.

[18] Wang Z., Oates T. (2015). Imaging time-series to improve classification and imputation. arXiv.

[19] Eckmann J.P., Kamphorst S.O., Ruelle D. (1987). Recurrence plots of dynamical systems. Europhys. Lett..

[20] Liang H., Zhong K., Song Y., Weng X., Wang X., Liu H., Xu L., Li Y., Zou W., Feng H. (2026). GAF-ResNet-MHSA: A novel transfer learning method for soil nutrient prediction in small sample datasets. Spectrochim. Acta Part A Mol. Biomol. Spectrosc..

[21] Zhai M., Wang Z., Yu Y., Li H., Mo X., Zhao M., Li Y., Zha Z., Wu J. (2025). Detection of sub-healthy apples with moldy core using dimension converted Vis-NIR transmission spectra combined with explainable deep-shallow model. Food Control.

[22] Tan A., Wang H., Zuo Y., Zhao R., Ma W., He Y., Zhao Y. (2025). IFCNN-based fusion of GAF and MTF encoded near-infrared spectral images for quantitative analysis of microplastics. Spectrochim. Acta Part A Mol. Biomol. Spectrosc..

[23] Xie F., Chen X., Jing Y., Li M., Li J., Zhao L. (2025). Wine variety traceability by data fusion of near-infrared (NIR) spectroscopy and mid-infrared (MIR) spectroscopy combined with GAF and ResNet. Chemom. Intell. Lab. Syst..

[24] Zhu Y., Wang T., Li Z., Ni W., Zhang K., He T., Fu M., Zeng M., Yang J., Hu N. (2024). Gas Identification Using Electronic Nose via Gramian-Angular-Field-Based Image Conversion and Convolutional Neural Networks Architecture Search. Sens. Actuators B Chem..

[25] Xu Z.-Y., Chen S., Li J.-G., Yao H.-L., Zhou P.-Y., Chen S.-Q., Zhao H.-J., Bai Y.h., Zhao D.b. (2025). Research on rapid and non-destructive detection model for pork freshness based on dual-branch hyperspectral feature extraction network combined with machine learning. J. Food Compos. Anal..

[26] Ji S., Hao S., Cui J., Yuan J., Xuan H. (2025). Identification of syrup adulteration in wolfberry honey using CNN-CBAM-SVM combined with 1H NMR. Food Chem..

[27] Zhao K., Yang Y., Zhang Y., Song Y., Shen T. (2025). Acoustic-vibration detection of moldy pear core: A novel approach combining image coding and hybrid deep learning. Food Control.

[28] Taheri-Garavand A., Fatahi S., Shahbazi F., de la Guardia M. (2019). A nondestructive intelligent approach to real-time evaluation of chicken meat freshness based on computer vision technique. J. Food Process Eng..

[29] Asmara R.A., Hasanah Q., Rahutomo F., Rohadi E., Siradjuddin I., Ronilaya F., Handayani A.N. (2018). Chicken meat freshness identification using colors and textures feature. Proceedings of the 2018 Joint 7th International Conference on Informatics, Electronics & Vision (ICIEV) and 2018 2nd International Conference on Imaging, Vision & Pattern Recognition (icIVPR), Kitakyushu, Japan, 25–29 June 2018.

[30] Sutarman S., Avianto D., Wibowo A.P. (2023). Vision-based chicken meat freshness recognition system using RGB color moment features and support vector machine. Sci. Inf. Technol. Lett..

[31] Li B., Ou-yang S.t., Li Y.b., Lu Y.j., Liu Y.d., Ou-yang A.g. (2025). Quantitative detection of beef freshness characterized by storage days based on hyperspectral imaging technology combined with physicochemical indexes. J. Food Compos. Anal..

[32] Shao Y., Shi Y., Wang K., Li F., Zhou G., Xuan G. (2023). Detection of small yellow croaker freshness by hyperspectral imaging. J. Food Compos. Anal..

[33] Wan G., Fan S., Liu G., He J., Wang W., Li Y., Cheng L., Ma C., Guo M. (2023). Fusion of spectra and texture data of hyperspectral imaging for prediction of myoglobin content in nitrite-cured mutton. Food Control.

[34] Dong F., Niu Y., Bi Y., Hao J., Wang S. (2024). Fusion of spectra and texture features of hyperspectral imaging for quantification and visualization of characteristic amino acid contents in beef. LWT.

[35] (2016). National Food Safety Standard–Determination of Volatile Basic Nitrogen in Food.

[36] He K., Zhang X., Ren S., Sun J. Deep residual learning for image recognition. Proceedings of the IEEE Conference on Computer Vision and Pattern Recognition.

[37] Deng J., Dong W., Socher R., Li L.J., Li K., Fei-Fei L. (2009). Imagenet: A large-scale hierarchical image database. Proceedings of the 2009 IEEE Conference on Computer Vision and Pattern Recognition, Miami, FL, USA, 20–25 June 2009.

[38] Guyon I., Weston J., Barnhill S., Vapnik V. (2002). Gene selection for cancer classification using support vector machines. Mach. Learn..

[39] Chen X., Xu Y., Meng L., Chen X., Yuan L., Cai Q., Shi W., Huang G. (2020). Non-parametric partial least squares–discriminant analysis model based on sum of ranking difference algorithm for tea grade identification using electronic tongue data. Sens. Actuators B Chem..

[40] Lundberg S.M., Lee S.I. (2017). A unified approach to interpreting model predictions. Adv. Neural Inf. Process. Syst..

[41] Xu W., He Y., Li J., Zhou J., Xu E., Wang W., Liu D. (2023). Portable beef-freshness detection platform based on colorimetric sensor array technology and bionic algorithms for total volatile basic nitrogen (TVB-N) determination. Food Control.

[42] Guo L., Wang T., Wu Z., Wang J., Wang M., Cui Z., Ji S., Cai J., Xu C., Chen X. (2020). Portable food-freshness prediction platform based on colorimetric barcode combinatorics and deep convolutional neural networks. Adv. Mater..

[43] (2016). National Food Safety Standard–Fresh and Frozen Livestock and Poultry Products.

[44] Pegau W.S., Gray D., Zaneveld J.R.V. (1997). Absorption and attenuation of visible and near-infrared light in water: Dependence on temperature and salinity. Appl. Opt..

[45] Yang Q., Sun D.W., Cheng W. (2017). Development of simplified models for nondestructive hyperspectral imaging monitoring of TVB-N contents in cured meat during drying process. J. Food Eng..

[46] Leng T., Li F., Chen Y., Tang L., Xie J., Yu Q. (2021). Fast quantification of total volatile basic nitrogen (TVB-N) content in beef and pork by near-infrared spectroscopy: Comparison of SVR and PLS model. Meat Sci..

[47] Fisher R.A. (1936). The use of multiple measurements in taxonomic problems. Ann. Eugen..

[48] Iandola F.N., Han S., Moskewicz M.W., Ashraf K., Dally W.J., Keutzer K. (2016). SqueezeNet: AlexNet-level accuracy with 50x fewer parameters and. arXiv.

[49] Sandler M., Howard A., Zhu M., Zhmoginov A., Chen L.C. Mobilenetv2: Inverted residuals and linear bottlenecks. Proceedings of the 2018 IEEE/CVFIEEE Conference on Computer Vision and Pattern Recognition.

[50] Zhang J., Liu G., Li Y., Guo M., Pu F., Wang H. (2022). Rapid identification of lamb freshness grades using visible and near-infrared spectroscopy (Vis-NIR). J. Food Compos. Anal..

[51] Zou L., Liu W., Lei M., Yu X. (2021). An improved residual network for pork freshness detection using near-infrared spectroscopy. Entropy.

